# Expanding power and opportunity in public health education: the continuous learning for antiracist culture change fellowship program

**DOI:** 10.3389/fpubh.2026.1766034

**Published:** 2026-03-24

**Authors:** Jonathan E. Cohen, Karina Dominguez Gonzalez, Ugonna Ihenacho, Pooja Shah, Roberta McKean-Cowdin, Ricky N. Bluthenthal

**Affiliations:** Department of Population and Public Health Sciences, Keck School of Medicine, University of Southern California, Los Angeles, CA, United States

**Keywords:** antiracist leadership, antiracist pedagogy, curricular change, diversity, equity and inclusion, power, racial equity

## Abstract

**Introduction:**

There is growing student demand in graduate public health education for addressing racism as a determinant of health and educational opportunity. Antiracist pedagogy provides a framework for understanding power and opportunity in public health education and strengthening leadership capacity among faculty and staff.

**Methods:**

We created a 15-week fellowship program for public health faculty and staff to learn about antiracist frameworks and practices. The program combined asynchronous learning with individualized coaching, peer support, and group activities for fellows. Fellows designed personal contracts to use their learning to effect tangible antiracist changes in their work with students.

**Results:**

A total of 26 faculty and staff completed the fellowship over four cohorts from September 2023–May 2025. Pre-fellowship, post-fellowship, and one-year post-fellowship survey data revealed significant increases in fellows’ capacity to review course content, teaching methods, and other educational activities to reflect antiracist theory and practice (*p* = 0.001). Fellows also reported increased expertise, knowledge of review criteria, and preparedness (*p* < 0.001), and awareness of their strengths and weaknesses in antiracist pedagogy (*p* = 0.004), as well as increased ability to provide concrete examples of how antiracist pedagogy works in practice (*p* = 0.003) and to identify specific tools, experts, and resources in antiracist theory and practice (*p* < 0.001). We observed fewer or less durable changes in other areas, such as fellows’ self-perceived contribution to a culture of self-reflection, learning and continuous improvement, fellows reporting having specific ideas for how to incorporate antiracism into public health education, or fellows being actively involved in colleagues’ and students’ learning and skill development in antiracism.

**Discussion:**

A cohort-based fellowship combining asynchronous learning and coaching should be considered among the growing number of options for integrating antiracism into public health education. Other universities interested in this model should also take into account lessons learned regarding the length of the program, the degree of independence expected from fellows, participant attrition, and resources available to implement fellowship projects.

## Introduction

In recent years, U.S. schools, departments, and programs of public health have encountered increasing demands from students and faculty to address racism as a key determinant of health and to integrate antiracist frameworks into educational practices (1–3). These demands reflect recognition that public health curricula have historically upheld racialized structures, both implicitly through adherence to Eurocentric norms and explicitly by omitting critical analysis of racism as a system of power and oppression. Empirical studies indicate that the Movement for Black Lives uprisings prompted greater scrutiny of how higher education perpetuates or challenges systemic inequities. Following the 2020 racial reckoning, students nationwide urged universities to move beyond symbolic gestures and implement substantive structural and culture reforms to advance equity and justice (4–7). This underscores the necessity for academic institutions, particularly in public health, to acknowledge their histories of racial inequity and to respond to the explicit demands of contemporary students engaged in a significant racial justice movement.

At the Keck School of Medicine (KSOM) of the University of Southern California (USC), students in the Department of Population and Public Health Sciences (DPPHS) identified a need for faculty and staff to enhance their competence in antiracist pedagogy, advising, and institutional decision-making. This was based on a systemic racism survey designed by three student groups in 2021 to gauge the students’ beliefs, priorities and desires for future directions in antiracist policy and curricula in DPPHS. Prior studies also indicate that such challenges are widespread across academic institutions. Numerous institutions have revised curricula and redesigned learning environments to address racism and structural inequities. For example, Hassen et al. (8) describe a public health program that engaged faculty, students, and administrators in examining practices, identifying systemic racism, and embedding antiracist content across courses. At USC, KSOM introduced a Health Justice and Systems of Care track as a required longitudinal course for undergraduate medical students, including topics such as racism in medicine, health inequities, and health systems science (9). Although such efforts indicate that reform is achievable, they also reveal the limitations of short-term interventions. Similar efforts across the nation underscore the growing importance of developing antiracist competencies in teaching, advising, and program design, with scholars documenting systematic culture reforms, rising student activism, and the adoption of antiracist competencies across Schools of Public Health (10, 11). Despite these advances, significant gaps remain: many initiatives rely on episodic workshops or course-specific revisions that lack the longitudinal structures necessary for sustained cultural change (1, 2).

The Continuous Learning for AntiRacist Culture Change (CLARCC) Fellowship was established to address these challenges through a sustained, cohort-based approach model for faculty and staff development. CLARCC is a 15-week, cohort-based faculty and staff development fellowship that integrates asynchronous learning, coaching, peer support, and applied antiracist practice. In contrast to programs that rely on episodic workshops or isolated course modifications, CLARCC provides continuous, collaborative development over a 15-week period, incorporating coaching, peer learning, and reflection. This model seeks to achieve lasting change by fostering individual growth and collective responsibility, thereby promoting departmental transformation. This initiative also represents the first time in Department history that staff have been included in a fellowship, challenging the norm of offering such programs solely to faculty and students and recognizing the essential role staff play in shaping student experience and departmental culture. Informed by the 2021 USC Racial Equity, Diversity and Inclusion (REDI) Task Force recommendations, CLARCC integrates ongoing learning, self-assessment, individualized coaching, and practical application of antiracist frameworks. The CLARCC Fellowship is anchored in an antiracist theory of change that recognizes racism as a structural system embedded in the policies, pedagogical norms, and institutional cultures that shape public health education (1, 2, 10).

**Figure 1:**
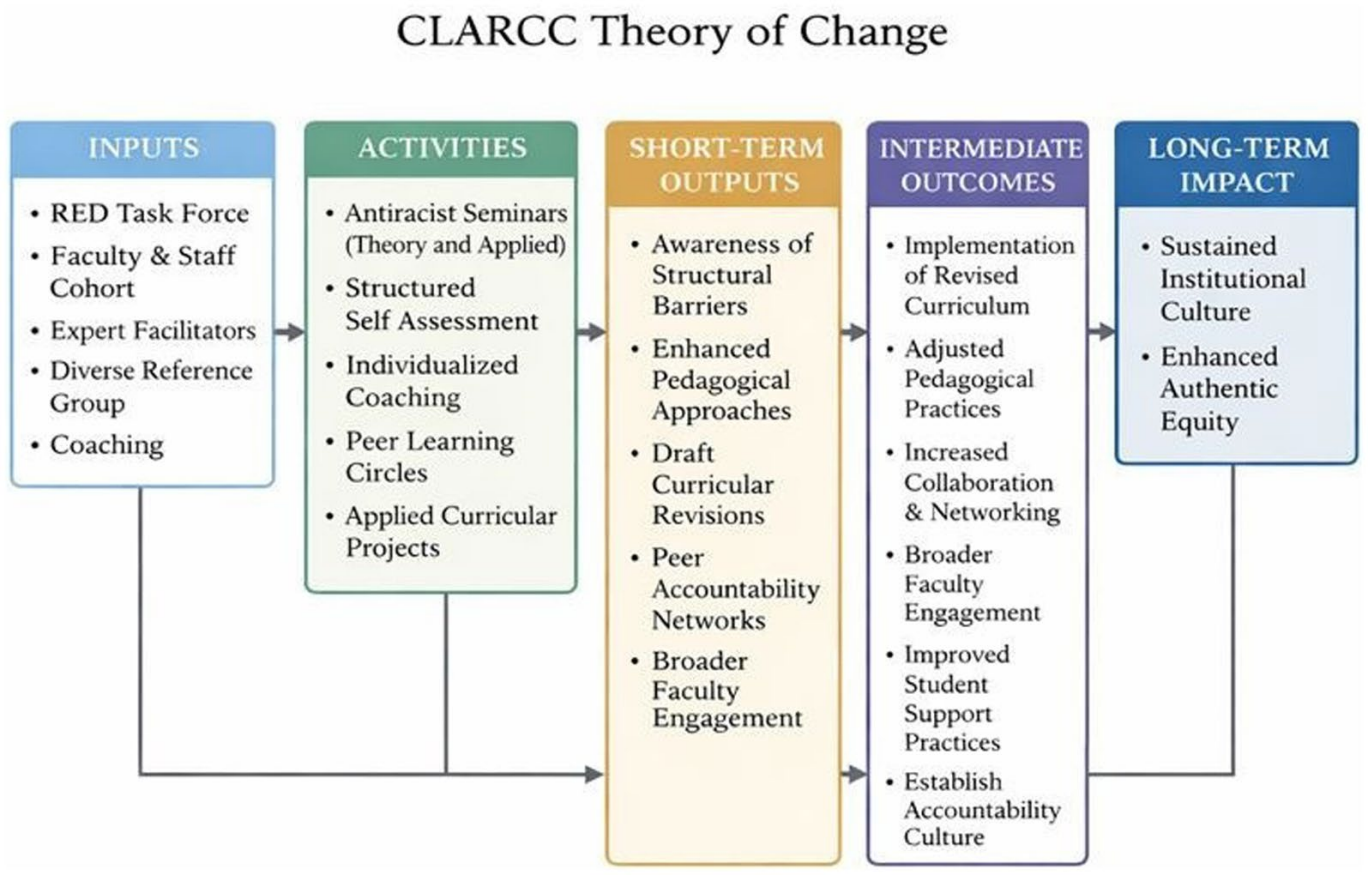
CLARCC Fellowship Theory of Change. Conceptual framework showing how fellowship inputs and activities produce short-term learning outputs, intermediate changes in pedagogical practice and collaboration, and long-term impacts on institutional culture and equity.

**Figure 2:**
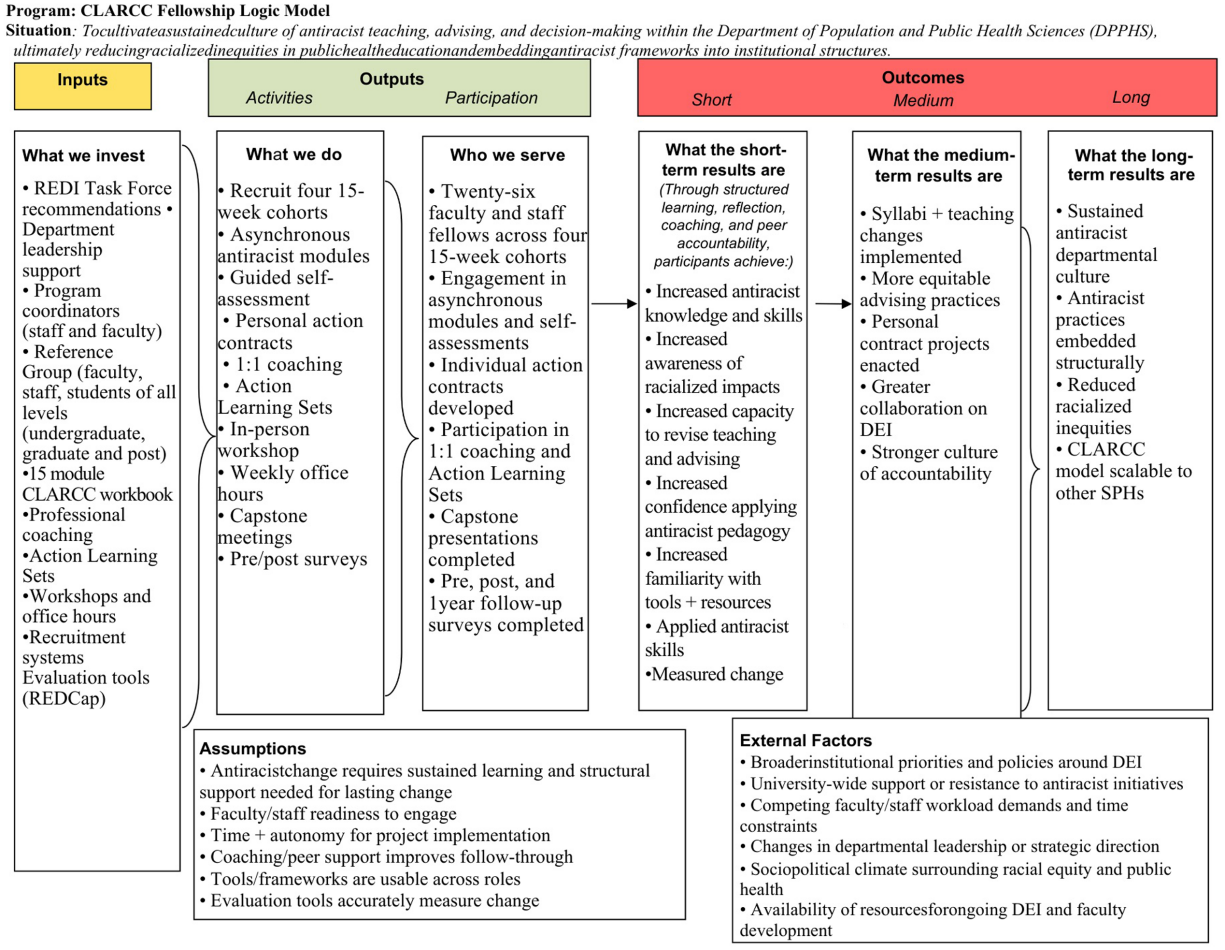
CLARCC Fellowship Logic Model. Conceptual framework depicting program inputs, fellowship activities, and participant engagement leading to short-term learning outcomes, medium-term pedagogical and institutional practice changes, and long-term impacts on departmental culture and equity.

The Theory of Change diagram (Figure 1) and accompanying Logic Model (Figure 2) demonstrate that meaningful transformation is achieved by aligning specific *inputs*, such as an antiracist curriculum, trained facilitators, and a structured learning arc, with intentional *activities* that foster continuous practice. These activities encompass critical antiracist instruction, self-assessment, individualized coaching, and peer learning. Existing literature indicates that short-term workshops rarely yield sustained outcomes and often do not alter deeply rooted racialized practices (8, 11). In contrast, sustained, cohort-based models that integrate accountability, reflection, and applied practice are more likely to produce measurable *outputs*, including increased awareness and enhanced pedagogical skills, as well as *intermediate outcomes* such as curricular revision, equitable advising practices, and greater confidence in addressing racialized dynamics (3, 7). CLARCC’s Theory of Change and Logic Model explicitly connect these stages to long-term *impact by* cultivating a departmental culture of continuous learning and accountability, embedding antiracist frameworks into institutional structures, and ultimately reducing racialized inequities in the educational environment. The Theory of Change and Logic Model thus provide a conceptual framework for understanding how structurally supported individual behavior change can scale into meaningful departmental and institutional transformation.

Building upon this conceptual foundation, the Department initiated a systematic, multi-phase process to design CLARCC’s structure, develop its curricular content, and establish a comprehensive evaluation strategy.

## Methods

### Program conceptualization

The development of CLARCC followed the 2021 report of the USC REDI Task Force, in particular its recommendations to integrate racial equity across the curriculum and to provide opportunities for staff and faculty to experience professionally led anti-racist trainings. A faculty coordinator (JEC) and staff coordinator (KDG) with experience leading DEI initiatives both inside and outside USC developed a concept note for an initial 18-month initiative from January 2023 – June 2024. Two additional co-authors (UI and PS) participated as Reference Group members to guide the program (see below), and the remaining two co-authors (RMC and RNB) contributed faculty oversight. The program aimed to:

1. Foster a culture of self-reflection, learning and continuous improvement to support antiracist culture change in the DPPHS.
2. Strengthen the capacities of DPPHS faculty to review course content, teaching methods, and other educational activities to reflect antiracist theory and practice
3. Document and demonstrate successful experiences in adapting course content, teaching methods, and other educational opportunities to antiracist principles
4. Build consensus and momentum across DPPHS for additional efforts to become a model of antiracist education, such as additional required courses or concentrations, additional professional development opportunities for faculty, and new recruitment.

The original concept note envisioned two learning cohorts of 6–12 faculty and staff members participating in a process of *facilitated self-assessment* and the development of *personal contracts* over a 12-month period. Facilitated self-assessment referred to the completion of surveys and exercises designed to help faculty and staff identify their own antiracist behaviors and their knowledge of antiracist frameworks that inform work with students. Personal contracts referred to the development of an individual project that would incorporate antiracist principles into the faculty or staff member’s ongoing work with students, whether in the classroom or elsewhere. Each of these activities would be supported by 1–1 coaching, peer learning among cohort members, cohort-wide activities, and ongoing engagement with students. The coordinators hypothesized that the combination of these activities would help to build a *culture of continuous and mutual learning* within the cohorts and eventually the department. This culture shift would complement and reinforce related efforts at USC to integrate racial equity into the student curriculum, such as substantive review of course syllabi.

### Program development

Following approval of the concept note by Departmental leadership, a seven-member Reference Group was formed to advise the project. Two co-authors (UI and PS) participated in this group. Among the coordinators (JEC and KDG) and Reference Group members, there was high representation of groups that have experienced institutional racism and intersecting forms of discrimination, including Black, Hispanic, South Asian, and LGBTQ+ persons, as well as persons with disabilities. The Reference Group was chaired by the faculty and staff coordinator and included two faculty members, two Masters students, and one postdoctoral student who had also completed her doctoral training in the DPPHS. Reference Group members were recruited through an open call for expressions of interest, advertised through the department’s division leadership. The tasks of the Reference Group were to (1) strengthen the conceptual framework for the program by validating and deepening the definition of antiracist culture change; (2) advise the program’s coordinators on the creation of a workbook that would guide the program experience of cohort members; and (3) advise the coordinators on a framework for evaluating the impact of the program on cohort members. Over a period of 6 months, the Reference Group carried out these tasks through a series of discussions and interactive exercises, including sharing personal stories of exclusion and inclusion, interviewing antiracism experts from outside USC, reviewing literature of existing antiracism programs in public health education, and exchanging antiracism self-assessment tools. It was in consultation with the Reference Group that the program was conceptualized as a “fellowship” in order to lend academic prestige to the process of committing to antiracist learning. Reference Group members also created the program’s evaluation framework through a discussion of what kinds of indicators would best illustrate progress toward the fellowship’s goals in our institutional context. One Reference Group member conducted a desk review of efforts to embed antiracism in American public health education, and was not able to find a comparable program to CLARCC in terms of the combination of asynchronous learning, coaching, peer support, group activities, and a personal contract integrated into a cohort-based fellowship experience.

Over the summer of 2023, the faculty coordinator drafted the workbook and evaluation framework for the program and incorporated comments from the staff coordinator and Reference Group members. (The evaluation framework is described further under “Results,” below.) The workbook was designed as 15 modules to be independently completed by fellows over a 15-week period, supported by enrichment activities (see below). Drawing on Ganz’s (12) public narrative framework, it introduces fellows to antiracist concepts through exploration of their “story of self” (Modules 1–5), “story of us” (Modules 6–10) and “story of now” (Modules 11–15). The “story of self” modules were designed to strengthen fellows’ ability to identify power dynamics in educational settings by prompting self-reflection on their individual anti-bias and antiracist attitudes. The “story of us” modules were designed to inspire fellows to commit to making a tangible antiracist change in their institution by introducing them to concrete examples of antiracist pedagogy and practice. Finally, the “story of now” modules were designed to stimulate fellows’ thinking and learning as they effect change by introducing them to important ideas and trends shaping antiracist work today.

Interspersed throughout the 15-week/15-module Workbook were activities that sought to enrich the fellows’ asynchronous individual learning through one-on-one coaching, peer support, and cohort-wide learning during weekly office hours. Each fellow was provided with five free hours of professional coaching during the 15-week period, and the Workbook prompted them to distribute these hours over the three modules. Coaches were sourced through Change Matrix, an organization contracted by the project sponsor to support grantees, and were contracted to provide five coaching hours to each fellow. The content of coaching was left entirely to the discretion of the fellows and their coaches, and fellows were not asked to report on this aspect of the program. The fellows were divided into small groups and prompted to schedule group meetings using an “Action Learning Set” methodology, whereby fellows take turns acting as mentors for each other using a structured format of short presentations and open-ended questions (13). Following completion of the first module, the full cohort met in-person to deepen their ability to identify power dynamics through an interactive role play exercise and share initial thinking about their personal contracts. Each fellow also met individually with both the staff and faculty coordinators to review a personal contract outlining specific goals and commitments for the duration of the fellowship. This individualized approach ensured accountability and enabled participants to integrate the fellowship experience into their professional practice in meaningful, personalized ways. Finally, the staff coordinator convened weekly “office hours” on Zoom for any fellow who wanted to check in, ask questions, and share feedback about the program and their experience.

### Cohort recruitment

Beginning in the summer of 2023, four cohorts of fellows were recruited to complete the program over four successive 15-week periods from September–December 2023 (Cohort 1), January–April 2024 (Cohort 2), September–December 2024 (Cohort 3), and January–April 2025 (Cohort 4). Enrollment in the CLARCC Fellowship was initially limited to faculty and staff within the DPPHS. Recruitment was opened to any faculty or staff member of the DPPHS and occurred through email announcements, faculty and staff listservs, and departmental newsletters approximately 2 months before the launch of each cohort. A program website was designed and launched to provide information and an application portal for interested faculty and staff, and a virtual “town hall” was convened before the first cohort to answer questions. Applications were open for a fixed period, during which applicants completed a structured form capturing their reflections on antiracist theory, prior involvement in diversity, equity, and inclusion (DEI) work, and motivation for participating in the fellowship. The program did not use formal eligibility criteria; instead, participation was intentionally open to both faculty and staff to promote equitable access to antiracist professional development.

Following a review of applications by the coordinators for completeness and clarity, all applicants were accepted. Cohort sizes ranged from 4 to 12 participants, reflecting the voluntary nature of the program and varying levels of interest across recruitment cycles. By the second cohort, interest expanded beyond the department, drawing applicants from across the Keck School of Medicine. To accommodate this broader demand, all eligible applicants were included in the program. As a voluntary program, the evaluation would reflect an inherent selection bias toward participants with an intrinsic motivation to engage in antiracist work. However, fellows’ applications revealed diverse backgrounds, skill levels, and motivations, suggesting that the program succeeded in reaching people who would not normally or otherwise engage in antiracist work. A review of fellowship application responses offers additional context regarding participant characteristics and motivations for enrolling in the CLARCC Fellowship. Application materials show that fellows held a variety of professional roles within the department and school, including instructional faculty, research faculty, student services staff, and administrative professionals, with differing lengths of tenure and institutional responsibilities. Applicants reported varying levels of prior engagement with antiracist or DEI work, ranging from extensive involvement in equity-focused initiatives to limited prior exposure and a desire to develop foundational understanding and practical skills. Many fellows cited personal and professional motivations rooted in lived experience, commitments to supporting students more equitably, or recognition of gaps in their own training. Collectively, these application data indicate that, despite voluntary participation, the fellowship attracted individuals with diverse backgrounds, readiness levels, and entry points into antiracist practice.

Following the completion of Cohorts 1 and 2 (in August 2024) and Cohorts 3 and 4 (in August 2025), a capstone meeting was convened for fellows who had completed the program in the preceding year. The goals of the each capstone meeting were to (1) share progress on personal contracts and exchange feedback among fellows and with other faculty and staff working on Departmental DEI initiatives; (2) foster shared responsibility among fellows, other faculty and staff working on Departmental DEI initiatives, and Departmental leadership for project execution going forward; and (3) identify opportunities to improve and expand the fellowship program based on pre- and post-fellowship survey data. To meet these goals, fellows shared short work-in-progress presentations of their personal contracts and engaged in dialogue with Departmental leadership about their projects and fellowship experience. At the first capstone meeting, fellows also discussed questions from the Evaluation Framework in small groups. Fellows did not receive specific ongoing support following the 15-week fellowship period and capstone meeting, however the process strengthened their relationships with and access to senior faculty who could help them further implement and promote their personal contracts. Fellows did not participate in the writing of this article, however their survey responses form the basis for the program evaluation.

### Data collection and analysis

To track progress and evaluate the fellowship’s impact, participants completed surveys assessing knowledge, attitudes, and self-reported competencies related to antiracist frameworks and DEI engagement. The evaluation aimed to assess (1) changes in fellows’ antiracist knowledge, attitudes, and self reported competencies over time; and (2) the feasibility, acceptability, and perceived institutional impact of the CLARCC Fellowship. Surveys were administered across three timepoints: pre-fellowship surveys in the first week of the fellowship program; a post-fellowship survey at completion of the program, and a 1-year post fellowship survey administered 1-year after completing the program.

Survey questions were designed to assess the four fellowship goals: (1) To foster a culture of self-reflection, learning and continuous improvement to support antiracist curricular change in the DPPHS; (2) To strengthen the capacities of DPPHS faculty to review course content, teaching methods, and other educational activities to reflect antiracist theory and practice; (3) To document and demonstrate successful experiences in adapting course content, teaching methods, and other educational opportunities to antiracist principle; (4) To build consensus and momentum across DPPHS for additional efforts to become a model of antiracist education, such as additional required courses or concentrations, additional professional development opportunities for faculty, new recruitment, etc. Three questions were developed for each goal, derived from a meeting of the Reference Group about which indicators would best illustrate progress in our institutional context. Limitations of this approach included the fact that the survey questions were not derived from published literature, and that some questions could have been categorized under multiple goals. Study data were collected and managed using REDCap electronic data capture tools hosted at USC (14).

As of December 2025, cohorts 1 and 2 had been invited to complete the 1-year post-fellowship survey and therefore make up the analytic sample for this assessment. Responses were provided on a 5-point Likert scale and evaluated as continuous measures. Among cohort 1 and 2, we used a mixed-effects linear regression model to evaluate changes in the survey measures across the 3 timepoints. The model included fixed effects for time (as a categorical variable) and cohort. We specified a random intercept for each participant to account for the correlation of repeated measurements within individuals. Model parameters were estimated using restricted maximum likelihood (REML). Survey scores were reported as cohort-adjusted means and 95% confidence intervals. We evaluated the overall effect of the fellowship over time using the Wald test. In a supplemental sensitivity analysis, we summarized data from the pre-fellowship and post-fellowship surveys across all 4 cohorts as means with standard deviations and used paired t-tests to test for differences in survey scores. All tests were assessed at a significance level of *p* < 0.05. Analyses were performed using Stata version 15.1 (StataCorp LLC, College Station, TX).

## Results

### Enrollment and cohort formation

A total of 12 faculty and 14 staff enrolled in the fellowship program over four cohorts. This disaggregated into 7 faculty and 3 staff in cohort 1, 4 faculty and 5 staff in cohort 2; 4 staff in cohort 3; and 1 faculty and 2 staff in cohort 2. These trends point to a decline in the faculty-to-staff ratio over the course of the program, perhaps suggesting that the demand for the program was more finite among faculty or that it took longer for staff to seek out the program than faculty. While it is difficult to estimate the percentage of total faculty and staff who enrolled given that total numbers shifted over the four cohorts, approximately 10% of faculty and 7% of staff in the department participated over the four cohorts.

### Participants and survey response rates

In total, 26 faculty and staff members participated in the fellowship. Of the 26 fellows, 24 (92.3%) completed the pre-fellowship survey and 17 (65.4%) completed the post-fellowship survey (Supplementary Table 1). Cohort 1 and 2 consisted of 19 fellows and of those fellows, 7 (36.8%) completed the 1-year post-fellowship survey.

### Goal 1: no changes in self-reflection and continuous improvement

Across the 7 prompts to assess the fellows self-efficacy and beliefs about fostering a culture of self-reflection, learning and continuous improvement to support antiracist curricular change, we observed mid-range mean scores that did not statistically significantly change over time (*p* > 0.05 for each; Table 1).

**Table 1:** CLARCC Fellows’ knowledge, attitudes, and self-reported competencies as reported across the three evaluation timepoints among Cohorts 1 and 2.

Question text	Scale	Pre-fellowship	Post-fellowship	1-Year Post-fellowship	Pre- vs post- fellowship		Pre- vs 1-year post-fellowship	Over follow-up^2^
		Mean (95% CI)^1^	Mean (95% CI)^1^	Mean (95% CI)^1^	*p*-value^2^		*p*-value^2^	
*Goal 1: To foster a culture of self-reflection, learning and continuous improvement to support antiracist curricular change in DPPHS.*								
I am actively involved in my colleagues’ and students’ learning and skill development in antiracism	0, Never | 1, Rarely | 2, Sometimes | 3, Frequently | 4, Always		2.28 (1.79–2.77)	2.87 (2.32–3.41)	2.39 (1.71–3.07)	**0.03**	0.75	0.08
I set an example of antiracism for my colleagues and students by learning and growing myself	0, Never | 1, Rarely | 2, Sometimes | 3, Frequently | 4, Always		2.83 (2.44–3.22)	3.06 (2.63–3.49)	3.00 (2.48–3.53)	0.23	0.48	0.46
I share and learn about antiracism with others through discussions and collaborative work	0, Never | 1, Rarely | 2, Sometimes | 3, Frequently | 4, Always		2.45 (1.98–2.92)	2.83 (2.32–3.34)	2.97 (2.35–3.59)	0.09	0.07	0.10
I have access to resources and development opportunities to become a better antiracist educator	0, Never | 1, Rarely | 2, Sometimes | 3, Frequently | 4, Always		2.17 (1.70–2.65)	2.79 (2.26–3.32)	2.56 (1.89–3.24)	**0.02**	0.25	0.07
I possess the capacity and motivation to learn about how to become a better antiracist educator	0, Never | 1, Rarely | 2, Sometimes | 3, Frequently | 4, Always		3.10 (2.74–3.46)	3.28 (2.89–3.68)	2.86 (2.36–3.35)	0.33	0.32	0.23
How would you describe your colleagues’ and students’ awareness of and reactions to your participation in the CLARCC Fellowship?	0, Unaware | 1, Indifferent | 2, Curious | 3, Supportive | 4, I do not know | 5, Other		2.00 (1.24–2.77)	2.34 (1.45–3.23)	2.43 (1.24–3.62)	0.53	0.52	0.74
How would you describe your colleagues’ and students’ attitudes toward antiracist curricular change in general?	0, Unaware | 1, Indifferent | 2, Curious | 3, Supportive | 4, I do not know | 5, Other		2.52 (1.99–3.06)	3.07 (2.45–3.70)	2.25 (1.41–3.09)	0.16	0.57	0.19
*Goal 2: To strengthen the capacities of DPPHS faculty to review course content, teaching methods, and other educational activities to reflect antiracist theory and practice.*								
I have relevant experience reviewing course content, teaching methods, and other educational activities according to antiracist theory and practice	0, Definitely Not | 1, Unlikely | 2, Not Sure | 3, Most Likely | 4, Definitely		1.85 (1.33–2.37)	2.85 (2.26–3.45)	3.11 (2.33–3.90)	**0.003**	**0.003**	**0.001**
I know what criteria I would use to review course content, teaching methods, and other educational activities according to antiracist theory and practice	0, Definitely Not | 1, Unlikely | 2, Not Sure | 3, Most Likely | 4, Definitely		1.83 (1.32–2.33)	2.53 (1.96–3.11)	3.15 (2.40–3.90)	**0.03**	**0.001**	**0.002**
I am aware that I have inadvertently caused microaggressions or other racial harm as a result of not incorporating antiracism into my work with students	0, Definitely Not | 1, Unlikely | 2, Not Sure | 3, Most Likely | 4, Definitely		2.63 (2.23–3.04)	3.14 (2.71–3.57)	2.71 (2.21–3.21)	**0.001**	0.70	**0.005**
I am aware of my strengths and weaknesses when it comes to reviewing course content, teaching methods, and other educational activities according to antiracist theory and practice	0, Definitely Not | 1, Unlikely | 2, Not Sure | 3, Most Likely | 4, Definitely		2.25 (1.96–2.55)	2.79 (2.45–3.14)	3.02 (2.56–3.48)	**0.01**	**0.004**	**0.004**
My professional experience adequately prepares me to review course content, teaching methods, and other educational activities according to antiracist theory and practice	0, Definitely Not | 1, Unlikely | 2, Not Sure | 3, Most Likely | 4, Definitely		1.77 (1.30–2.24)	2.84 (2.29–3.38)	3.23 (2.50–3.95)	**0.001**	**<0.001**	**<0.001**
*Goal 3: To document and demonstrate successful experiences in adapting course content, teaching methods, and other educational opportunities to antiracist principles.*								
I know it is possible to integrate antiracism into public health education because I have seen it done	0, Strongly Disagree | 1, Somewhat Disagree | 2, Neither Disagree nor Agree | 3, Somewhat Agree | 4, Strongly Agree		2.88 (2.42–3.35)	3.43 (2.92–3.93)	3.33 (2.72–3.94)	**0.01**	0.11	**0.03**
I am able to provide a concrete example of what antiracist pedagogy in public health education looks like in practice	0, Strongly Disagree | 1, Somewhat Disagree | 2, Neither Disagree nor Agree | 3, Somewhat Agree | 4, Strongly Agree		1.96 (1.31–2.62)	2.88 (2.16–3.61)	3.35 (2.43–4.27)	**0.01**	**0.002**	**0.003**
I have specific ideas for how we can better integrate antiracist pedagogy into public health education at USC	0, Strongly Disagree | 1, Somewhat Disagree | 2, Neither Disagree nor Agree | 3, Somewhat Agree | 4, Strongly Agree		2.24 (1.69–2.79)	2.93 (2.30–3.55)	2.99 (2.17–3.80)	**0.045**	0.08	0.07
I am aware of specific tools, experts, and resources to guide me in integrating antiracism into public health education	0, Strongly Disagree | 1, Somewhat Disagree | 2, Neither Disagree nor Agree | 3, Somewhat Agree | 4, Strongly Agree		1.75 (1.13–2.36)	3.16 (2.47–3.84)	3.34 (2.43–4.25)	**<0.001**	**<0.001**	**<0.001**
I believe that my colleagues would be more willing to integrate antiracism into their educational work if only they saw it in practice	0, Strongly Disagree | 1, Somewhat Disagree | 2, Neither Disagree nor Agree | 3, Somewhat Agree | 4, Strongly Agree		3.00 (2.56–3.44)	3.10 (2.60–3.61)	2.81 (2.09–3.53)	0.73	0.63	0.78
*Goal 4: To build consensus and momentum across DPPHS for additional efforts to become a model of antiracist education, such as additional required courses or concentrations, additional professional development opportunities for faculty, new recruitment, etc.*								
Recruiting more faculty from underrepresented groups, including racial minorities	0, Priority 1 (higher) | 1, Priority 2 | 2, Priority 3 | 3, Priority 4 | 4, Priority 5 (lower)		1.28 (0.71–1.84)	1.34 (0.69–1.99)	2.90 (1.99–3.82)	0.86	**0.001**	**0.003**
Admitting more students from underrepresented groups, including racial minorities	0, Priority 1 (higher) | 1, Priority 2 | 2, Priority 3 | 3, Priority 4 | 4, Priority 5 (lower)		2.62 (2.05–3.20)	2.69 (2.01–3.38)	2.62 (1.63–3.62)	0.88	0.99	0.99
Changing the standards and criteria by which faculty and staff are evaluated and promoted	0, Priority 1 (higher) | 1, Priority 2 | 2, Priority 3 | 3, Priority 4 | 4, Priority 5 (lower)		2.44 (1.74–3.14)	2.35 (1.54–3.16)	2.40 (1.25–3.54)	0.86	0.95	0.98
Investing in culture change to create a more enabling environment for continuous antiracist learning	0, Priority 1 (higher) | 1, Priority 2 | 2, Priority 3 | 3, Priority 4 | 4, Priority 5 (lower)		1.78 (1.14–2.41)	2.22 (1.50–2.94)	0.84 (−0.14–1.83)	0.24	0.07	**0.03**
Scaling up faculty development programs like CLARCC across the entire Department or School	0, Priority 1 (higher) | 1, Priority 2 | 2, Priority 3 | 3, Priority 4 | 4, Priority 5 (lower)		1.89 (1.26–2.51)	1.43 (0.73–2.13)	1.61 (0.68–2.54)	0.18	0.54	0.40

^1^Reporting means (95% CI) calculated using the numeric survey scores in the “Scale” column. Bold values indicate a statistically significant difference (*p* < 0.05) in mean survey scores by a mixed-effects linear regression.

CI, confidence interval; CLARCC, Continuous Learning for Antiracist Culture Change; DPPHS, Department of Population and Public Health Sciences; USC, University of Southern California.

### Goal 2: an increased capacity to incorporate antiracist theory and practice was reported

We observed statistically significant increases in the mean survey score over time for each of the 5 prompts measured to assess Goal 2 signifying an increased awareness or capacity to review course content, teaching methods, and other educational activities to reflect antiracist theory and practice (Table 1). Mean scores increased across all three timepoints for each measure, except fellows reported a decrease in their level of awareness of microaggressions from the post-fellowship survey to the 1-year post fellowship survey.

### Goal 3: increased knowledge for adopting antiracist principles

The scores of fellows increased for three domains used to assess their knowledge and ability to adapt antiracist pedagogy into course content over time (Table 1); these differences reached statistical significance. Immediately post-fellowship, we found a statistically significant increase in fellows ability to identify specific ideas toward integrating antiracist pedagogy in their work with students compared to pre-fellowship (*p* = 0.045), and scores remained elevated but stable through the 1-year post fellowship assessment (Wald test *p* = 0.07).

### Goal 4: variation in responses about building momentum for antiracist education

In the time from the pre-fellowship survey to 1-year post fellowship, there was a decrease in the belief that the department would need to prioritize recruiting more faculty from underrepresented groups, including racial minorities to become a model of antiracist education (*p* < 0.003; Table 1). However, the fellows did report a need to prioritize investing in culture change to create a more enabling environment for continuous antiracist learning (*p* = 0.03). Although the score did not change significantly overtime, scaling up faculty development programs like CLARCC across the entire Department or School was ranked as a high priority across each timepoint (mean ranged from 1.43–1.89).

### Sensitivity analysis of pre- and post-fellowship scores across all cohorts

When we evaluated changes between pre- and post-fellowship scores across Cohorts 1–4 (Supplementary Table 2), the magnitude and direction of the results were similar to the primary results in the mixed-effects model when restricted to the first two data timepoints.

### Description of personal contracts

Of 26 fellows, 17 submitted a progress report about their personal contract before their assigned capstone meeting. The attrition may have been due to a variety of factors, including voluntary withdrawal from the program due to competing commitments, professional transition out of the department or school, or failure to submit a progress report despite stable participation in the program. The progress reports indicated that fellows chose diverse topics of their contracts, indicative of the wide range of antiracist interventions that fellows perceived could benefit the department. Broadly speaking, personal contracts could be grouped into four categories: revising curricula and teaching methods (7); creating an enabling DEI environment (4); addressing financial accessibility issues (2); supporting specific student populations (2); and improving recruitment and onboarding (2). These thematic categories illustrate how fellows translated antiracist learning into specific, practice-based interventions across curricular, interpersonal, and institutional domains. The range of contract topics indicates that fellows identified several actionable pathways for promoting antiracist culture change within the department, moving beyond individual teaching to address structural, financial, and organizational aspects of equity. Collectively, these personal contracts offer qualitative evidence of program impact that is not fully reflected in survey data.

## Discussion

Our experience with the CLARCC Fellowship sheds light on the nature and durability of changes that can result from a cohort-based antiracist professional development program consisting of asynchronous learning combined with coaching and peer support. The most durable effects of the program were on fellows’ perceived capacity to review course content, teaching methods, and other educational activities to reflect antiracist theory and practice (Goal 2). Compared to the pre-fellowship survey, both immediately following the fellowship and a year later, fellows reported increased expertise, knowledge of review criteria, preparedness (*p* = 0.001 and *p* < 0.001, respectively), and awareness of their strengths and weaknesses in this area (*p* = 0.01 and *p* = 0.004, respectively). Other changes that endured over a year following the program included an increase in fellows’ self-reported ability to provide a concrete example of how antiracist pedagogy works in practice (*p* = 0.003), as well as in their awareness of specific tools, experts, and resources in antiracist theory and practice (*p* < 0.001).

Other desired changes were observed immediately following the fellowship program but not at the 1-year-post follow-up. These included an increase in fellows reporting having specific ideas for how to incorporate antiracism into public health education, being aware of having caused microaggressions or other racial harm as a result of not integrating antiracism into work with students, being actively involved in colleagues’ and students’ learning and skill development in antiracism, and having access to resources and development opportunities to become a better antiracist educator. While further study would be needed to understand why some changes endured longer than others, it stands to reason that some of the foregoing changes would be more “fresh in one’s mind” immediately following a fellowship program than they would be a year later. These data point to opportunities to tailor refresher programs and post-fellowship interventions to specific outcomes that are less likely to endure without them.

One significant change, namely, the priority fellows accorded to recruiting more faculty from underrepresented groups, including racial minorities, was observed a year following the fellowship but not immediately afterwards. There are many possible explanations for the delay in this change, including external factors only indirectly related to fellows’ participation in the program. Overall, the fellowship did not have a significant effect on fellows’ prioritization of various options for making the department more of a model of antiracist education. Nor did the fellowship appear to significantly impact fellows’ self-perceived contribution to a culture of self-reflection, learning and continuous improvement, for example whether they think they set an example of antiracism for fellow colleagues and students, share and learn about antiracism with others, or possess the capacity and motivation to learn how to become a better antiracist educator. Fellows’ perception of their colleagues’ and students’ awareness of and reaction to their participation in the fellowship program and attitudes toward antiracism in general also remained significantly unchanged.

There are at least two important explanations for the lack of observable changes in some of the areas where change was sought. The first is that a fellowship program such as CLARCC is by itself insufficient to influence department-wide culture change. The literature and USC’s own experience suggest multiple additional strategies that could be used in combination with a cohort-based faculty-staff fellowship program, such as student mentorship programs, broad curricular reforms, faculty and staff training seminars, and student fellowships (see, e.g., (11, 15–19)). Universities considering replicating CLARCC should situate it within a broader strategy for effecting antiracist change and account for this in their evaluation of each effort. The second explanation is deficiencies in CLARCC itself, especially given that it was a new program. Some opportunities for improvement that we identified include increasing the number of in-person interactions among fellows, allowing more time for fellows to complete the program, setting out clearer expectations for progress on coaching and personal contracts, integrating more continuous evaluation into the program, and digitizing the CLARCC workbook into a series of interactive online modules to facilitate asynchronous learning. Some of these improvements have cost implications, just as CLARCC itself is reliant on financial investment in staff and faculty time, coaching, and convening costs.

While we primarily measured changes at the individual level, changes at the interpersonal and institutional level could also be observed from the program. An example of this is the variety of personal contracts that fellows proposed and discussed with departmental leadership as part of program participation. As noted above, these contracts covered a wide variety of topics, such as revising curricula and teaching methods (e.g., revising course syllabi using antiracist criteria, experimenting with gradeless learning), creating an enabling DEI environment (e.g., improving employee resource groups, creating opportunities for staff connection); addressing financial accessibility issues (e.g., financial barriers to study abroad programs, examining expectations on student volunteers), increasing opportunity for specific populations of students (e.g., pregnant or expecting parents, undocumented migrants), and improving recruitment and onboarding (e.g., equitable recruitment of PhD students, teaching the “hidden curriculum”). The capacity and institutional focus of these contracts suggest that fellows translated individual learning into concrete actions that extended beyond self-reflection alone. However, these forms of change are difficult to capture through survey-based measures, stressing the need for qualitative and other approaches in future assessments of departmental culture change. The support and creative input that fellows extended to one another in defining and developing these projects was one of the most gratifying to notable aspects of the program.

There are important limitations on our evaluation findings that point to opportunities for future research and program expansion. First is the decreasing size of each successive cohort, likely due to the finite number of faculty and staff interested in participating in an unpaid antiracism fellowship program. Second is the attrition in survey participation, particularly for the one-year follow-up survey. Third is the relative lack of qualitative data to supplement survey findings, which limits our ability to explain and address certain changes or lack thereof. Although a focus-group exercise was conducted at the 2024 capstone meeting to gather input for program improvement, the data from these discussions were not collected and analyzed for research and evaluation purposes. Additional improvements in study design (including opportunities for future study) include evaluating the outcomes and impact of fellows’ personal contracts and enrolling additional cohorts.

## Conclusion

The CLARCC Fellowship is one of the few programs to our knowledge that uses a combination of cohort-based asynchronous learning and individual and peer-to-peer coaching to effect a culture change toward antiracist public health education. Our experience shows that, without compensation, a significant number of public health faculty and staff are willing to participate in such a program and see it to completion, and that their participation corresponds to statistically significant changes in indicators associated with antiracist culture change. At the same time, the evaluation findings point out the difficulty in achieving and measuring some aspects of culture change. While individual level capacity building appeared more significant, changes in self-reflection, collective responsibility, and institutional transformation were less consistently captured by survey measures alone. The personal contract commitments by fellows suggest that meaningful interpersonal and institutional action was initiated, but these type of change efforts require more longitudinal evaluation using mixed methods.

Our experience with the CLARCC fellowship generated lessons for other schools and programs of public health wishing to adapt the program, as well as pointing to future directions for the fellowship. We recommend that other schools emphasize the independent and asynchronous nature of the program in order to manage expectations about the amount of in-person instructional time. We also recommend that other schools clarify that personal contracts could take months or years to implement following the program, and that a sufficient outcome for a 15-week fellowships is simply a contract—a promise to oneself—along with a progress report. Alternatively, other schools may wish to consider lengthening the fellowship period in order to allow more time for completing the Workbook exercises and developing the contract. Among the enrichment activities, evidence from office hours with fellows suggest that the coaching was most popular. The peer mentorship through Action Learning Sets proved difficult to implement without close oversight by program coordinators, suggesting that a deeper orientation on this enrichment component would have increased its utility. All three of the group activities—the onboarding meeting, mid-fellowship meeting, and capttone meeting—proved highly popular with fellows and should be included in any iteration of the program. For schools interested in learning more from CLARCC, the complete fellowship Workbook, meeting agendas, survey results, and other administrative documents are available on request to the authors.

In addition to potentially enrolling additional cohorts, future directions for the CLARCC fellowship include continued research and evaluation, Workbook development, and support for fellows’ personal contracts. Once one-year post-fellowship data are available from all four cohorts, we will analyze survey results along with qualitative data to develop a complete picture of the fellowship outcomes and, to the extent this is revealed by qualitative data, the reasons behind these outcomes. We are also in the process of digitizing the CLARCC Workbook (currently an MS Word document) into an interactive online course using instructional software such as Canvas or Brightspace, as this will increase its accessibility and functionality and permit fellows to more seamlessly collaborate with one another online. Finally, through existing DEI structures we hope to support former fellows in continuing to implement their personal contracts, including seeking grant support where this is needed. The ideas for institutional reform represented by the fellows’ personal contracts, as well as the buy-in for these ideas among departmental leadership as a result of the CLARCC capstone meetings, are a significant outcome of the program that points to the potential for structural change through an individual fellowship approach.

## Data Availability

The raw data supporting the conclusions of this article will be made available by the authors, without undue reservation.
