# Supplementary material for: Expanding power and opportunity in public health education: the continuous learning for antiracist culture change fellowship program
Source: Front Public Health. 2026 Mar 24;14:1766034. doi: 10.3389/fpubh.2026.1766034 (PMC13055629; doi:10.3389/fpubh.2026.1766034)
Supplement: Supplementary file 1 [file Table_1.docx]

Supplementary Table 1. Survey Response Rates by Cohort

|  | Total Population | Cohort 1 | Cohort 2 | Cohort 3 | Cohort 4 |
| --- | --- | --- | --- | --- | --- |
|  | N = 26 | N = 10 | N = 9 | N = 4 | N = 3 |
| N (%)^1^ |  |  |  |  |  |
| Pre-Fellowship | 24 (92.3) | 9 (90.0) | 9 (100) | 3 (75.0) | 3 (100) |
| Post-Fellowship | 17 (65.4) | 9 (90.0) | 4 (44.4) | 2 (50.0) | 2 (66.7) |
| 1-Year Post-Fellowship | 7 (36.8)^2^ | 4 (40.0) | 3 (33.3) | --- | --- |

^1^ All values are counts and column percentages unless otherwise noted.

^2^ Total population uses a denominator of N=19 for the 1-Year Post Fellowship survey response rate because as of December 2025, Cohorts 3 & 4 have not yet been invited to complete the survey.
