# Supplementary material for: Expanding power and opportunity in public health education: the continuous learning for antiracist culture change fellowship program
Source: Front Public Health. 2026 Mar 24;14:1766034. doi: 10.3389/fpubh.2026.1766034 (PMC13055629; doi:10.3389/fpubh.2026.1766034)
Supplement: Supplementary file 2 [file Table_2.docx]

Supplementary Table 2. Summary changes in responses from pre-to post-fellowship among Cohort 1-4

| **Question Text** | **Scale** | **Pre-Fellowship Mean (SD)^1^** | **Post-Fellowship Mean (SD)^1^** | **Paired t-Test p-value^2^** |
| --- | --- | --- | --- | --- |
|  |  | N=17 | N=17 |  |
| ***Goal 1: To foster a culture of self-reflection, learning and continuous improvement to support antiracist curricular change in DPPHS.*** | | | | |
| **I am actively involved in my colleagues' and students' learning and skill development in antiracism** | 0, Never \| 1, Rarely \| 2, Sometimes \| 3, Frequently \| 4, Always | 2.24 (1.44) | 2.82 (0.73) | **0.046** |
| **I set an example of antiracism for my colleagues and students by learning and growing myself** | 0, Never \| 1, Rarely \| 2, Sometimes \| 3, Frequently \| 4, Always | 2.88 (0.86) | 3.18 (0.64) | 0.06 |
| **I share and learn about antiracism with others through discussions and collaborative work** | 0, Never \| 1, Rarely \| 2, Sometimes \| 3, Frequently \| 4, Always | 2.47 (1.23) | 2.71 (0.77) | 0.36 |
| **I have access to resources and development opportunities to become a better antiracist educator** | 0, Never \| 1, Rarely \| 2, Sometimes \| 3, Frequently \| 4, Always | 2.24 (1.20) | 3.00 (0.79) | **0.01** |
| **I possess the capacity and motivation to learn about how to become a better antiracist educator** | 0, Never \| 1, Rarely \| 2, Sometimes \| 3, Frequently \| 4, Always | 2.94 (0.97) | 3.41 (0.62) | **0.04** |
| **How would you describe your colleagues' and students' awareness of and reactions to your participation in the CLARCC Fellowship?** | 0, Unaware \| 1, Indifferent \| 2, Curious \| 3, Supportive \| 4, I don't know \| 5, Other | 1.76 (2.08) | 2.29 (1.16) | 0.32 |
| **How would you describe your colleagues' and students' attitudes towards antiracist curricular change in general?** | 0, Unaware \| 1, Indifferent \| 2, Curious \| 3, Supportive \| 4, I don't know \| 5, Other | 2.35 (1.22) | 2.88 (0.99) | 0.16 |
| ***Goal 2: To strengthen the capacities of DPPHS faculty to review course content, teaching methods, and other educational activities to reflect antiracist theory and practice.*** | | | | |
| **I have relevant experience reviewing course content, teaching methods, and other educational activities according to antiracist theory and practice** | 0, Definitely Not \| 1, Unlikely \| 2, Not Sure \| 3, Most Likely \| 4, Definitely | 1.82 (1.19) | 2.88 (0.86) | **0.002** |
| **I know what criteria I would use to review course content, teaching methods, and other educational activities according to antiracist theory and practice** | 0, Definitely Not \| 1, Unlikely \| 2, Not Sure \| 3, Most Likely \| 4, Definitely | 1.65 (1.22) | 2.71 (1.05) | **0.002** |
| **I am aware that I have inadvertently caused microaggressions or other racial harm as a result of not incorporating antiracism into my work with students** | 0, Definitely Not \| 1, Unlikely \| 2, Not Sure \| 3, Most Likely \| 4, Definitely | 2.41 (0.94) | 3.06 (0.75) | **0.004** |
| **I am aware of my strengths and weaknesses when it comes to reviewing course content, teaching methods, and other educational activities according to antiracist theory and practice** | 0, Definitely Not \| 1, Unlikely \| 2, Not Sure \| 3, Most Likely \| 4, Definitely | 2.29 (0.77) | 3.00 (0.61) | **0.003** |
| **My professional experience adequately prepares me to review course content, teaching methods, and other educational activities according to antiracist theory and practice** | 0, Definitely Not \| 1, Unlikely \| 2, Not Sure \| 3, Most Likely \| 4, Definitely | 1.53 (1.23) | 2.82 (0.81) | **<0.001** |
| ***Goal 3: To document and demonstrate successful experiences in adapting course content, teaching methods, and other educational opportunities to antiracist principles.*** | | | | |
| **I know it is possible to integrate antiracism into public health education because I have seen it done** | 0, Strongly Disagree \| 1, Somewhat Disagree \| 2, Neither Disagree nor Agree \| 3, Somewhat Agree \| 4, Strongly Agree | 2.76 (1.09) | 3.35 (0.86) | **0.01** |
| **I am able to provide a concrete example of what antiracist pedagogy in public health education looks like in practice** | 0, Strongly Disagree \| 1, Somewhat Disagree \| 2, Neither Disagree nor Agree \| 3, Somewhat Agree \| 4, Strongly Agree | 2.00 (1.46) | 3.06 (1.03) | **0.003** |
| **I have specific ideas for how we can better integrate antiracist pedagogy into public health education at USC** | 0, Strongly Disagree \| 1, Somewhat Disagree \| 2, Neither Disagree nor Agree \| 3, Somewhat Agree \| 4, Strongly Agree | 2.06 (1.25) | 2.88 (1.05) | **0.01** |
| **I am aware of specific tools, experts, and resources to guide me in integrating antiracism into public health education** | 0, Strongly Disagree \| 1, Somewhat Disagree \| 2, Neither Disagree nor Agree \| 3, Somewhat Agree \| 4, Strongly Agree | 1.59 (1.33) | 3.18 (0.95) | **<0.001** |
| **I believe that my colleagues would be more willing to integrate antiracism into their educational work if only they saw it in practice** | 0, Strongly Disagree \| 1, Somewhat Disagree \| 2, Neither Disagree nor Agree \| 3, Somewhat Agree \| 4, Strongly Agree | 3.06 (0.97) | 3.12 (0.70) | 0.79 |
| ***Goal 4: To build consensus and momentum across DPPHS for additional efforts to become a model of antiracist education, such as additional required courses or concentrations, additional professional development opportunities for faculty, new recruitment, etc.*** | | | | |
| **Recruiting more faculty from underrepresented groups, including racial minorities** | 0, Priority 1 (higher) \| 1, Priority 2 \| 2, Priority 3 \| 3, Priority 4 \| 4, Priority 5 (lower) | 1.65 (1.32) | 1.71 (1.31) | 0.88 |
| **Admitting more students from underrepresented groups, including racial minorities** | 0, Priority 1 (higher) \| 1, Priority 2 \| 2, Priority 3 \| 3, Priority 4 \| 4, Priority 5 (lower) | 2.35 (1.32) | 2.65 (1.41) | 0.52 |
| **Changing the standards and criteria by which faculty and staff are evaluated and promoted** | 0, Priority 1 (higher) \| 1, Priority 2 \| 2, Priority 3 \| 3, Priority 4 \| 4, Priority 5 (lower) | 2.47 (1.37) | 2.24 (1.56) | 0.59 |
| **Investing in culture change to create a more enabling environment for continuous antiracist learning** | 0, Priority 1 (higher) \| 1, Priority 2 \| 2, Priority 3 \| 3, Priority 4 \| 4, Priority 5 (lower) | 1.65 (1.62) | 1.76 (1.35) | 0.81 |
| **Scaling up faculty development programs like CLARCC across the entire Department or School** | 0, Priority 1 (higher) \| 1, Priority 2 \| 2, Priority 3 \| 3, Priority 4 \| 4, Priority 5 (lower) | 1.88 (1.41) | 1.65 (1.37) | 0.54 |

^1^Reporting means (SD) calculated using the numeric survey scores in the "Scale" column.

^2^*p*<0.05 indicates a statistically significant difference in mean score for the pre-fellowship responses compared to the post-fellowship responses among participants that responded to both surveys by a paired t-test.

Abbreviations: CLARCC, Continuous Learning for Antiracist Culture Change; DPPHS, Department of Population and Public Health Sciences; SD, standard deviation; USC, University of Southern California.
